# Supplementary material for: Drug detoxification dynamics explain the postantibiotic effect
Source: Mol Syst Biol. 2017 Oct 23;13(10):948. doi: 10.15252/msb.20177723 (PMC5658699; doi:10.15252/msb.20177723)
Supplement: Supplementary file 1 — Appendix [file MSB-13-948-s001.pdf]

**Drug detoxification dynamics explain the postantibiotic effect**

**Appendix**

Jaydeep K. Srimani[1], Shuqiang Huang[2], Allison J. Lopatkin[1], and Lingchong You[1,3,4*]

[1]Department of Biomedical Engineering, Duke University

[2]Center for Synthetic Biology Engineering Research, Shenzhen Institute of Advanced Technology, Chinese Academy of Sciences

[3]Center for Genomic and Computational Biology, Duke University

[4]Department of Molecular Genetics and Microbiology, Duke University School of Medicine

*Correspondence should be addressed to Lingchong You (you@duke.edu), 101 Science Drive, Durham, NC 27708

## Table of Contents

Appendix Table S1. Full ODE model parameter values ........ 3
Appendix Table S2. Mechanism model parameter values ........ 4
Appendix Table S3. Descriptions of antibiotics used ........ 5
Appendix Figure S1. Fluorescence luminescence reporters accurately reflect density following antibiotic treatment ........ 6
Appendix Figure S2. Partial population death does not account for antibiotic dependent recovery time ........ 7
Appendix Figure S3. Recovery time is not dependent on initial cell density ........ 8
Appendix Figure S4. Correlation between $RT_{pop}$ and $RT_{cell}$ is robust to ribosome degradation rate .. 9
Appendix Figure S5. Recovery time as a predictive metric for periodic dosing efficacy ........ 10
Appendix Figure S6. [CCCP] inhibits efflux pump activity in a dose-dependent manner ........ 11
Appendix Figure S7. Efflux inhibition is effective at high degradation rates and strong positive feedback conditions ........ 12
Appendix Figure S8. Determining $IC_{50}$ values for various antibiotics ........ 13

**Appendix Table S1. Full ODE model parameter values**

| Parameter | Value | Description |
|---|---|---|
| $k_1$ | 0.2 $\mu Mmin^{-1}$ | Synthesis rate constant of [C] |
| $k_f$ | 2.5 $\mu M^{-1}min^{-1}$ | Binding rate constant between [A] and [C] |
| $k_b$ | 1.5 $min^{-1}$ | Dissociation rate constant of [CA] |
| $k_{in}$ | 7e-3 $min^{-1}$ | Antibiotic influx rate constant |
| $k_{out}$ | 1e-2 $min^{-1}$ | Antibiotic efflux rate constant |
| $k_d$ | 0.1 $min^{-1}$ | Antibiotic-mediated ribosome inactivation rate constant |
| $k_r$ | 0.02 $min^{-1}$ | Antibiotic-mediated ribosome turnover rate constant |
| $k_u$ | 0.1 $min^{-1}$ | Intrinsic ribosome turnover rate constant |
| $V_1$ | 0.2 µM | Half-maximal ribosome concentration |
| $\mu$ | 0.05 | Basal cell growth rate |
| $V$ | 1.5 µM | Half-maximal ribosome concentration for growth rate scaling |
| $N_m$ | 1 | Carrying capacity |
| $C_0$ | 1e-1 µM | Initial concentration of [C] |
| $A_{in,0}$ | 0 µM | Initial concentration of [$A_{in}$] |
| $CA_0$ | 0 µM | Initial concentration of [CA] |
| $CA'_0$ | 0 µM | Initial concentration of [CA’] |
| $N_0$ | 1e-1 | Initial concentration of [N] |

**Appendix Table S2. Mechanism model parameter values**

| Parameter | Value | Description |
|---|---|---|
| $k_0$ | 1.25e-3 μMmin$^{-1}$ | Constitutive synthesis rate constant of [T] |
| $k_t$ | 0.01 μMmin$^{-1}$ | Maximum synthesis rate of [T] due to positive feedback |
| $K_T$ | 0.5 μM | Half-maximal value of [T] |
| $k_f$ | 0.25 μM$^{-1}$min$^{-1}$ | Antibiotic-target binding rate constant |
| $k_b$ | 0.075 min$^{-1}$ | Dissociation rate constant |
| $d_T$ | 0.01 min$^{-1}$ | Degradation rate constant of [T] |
| $d_P$ | 0.01 min$^{-1}$ | Degradation rate constant of antibiotic-target complex |
| $k_{in}$ | 0.1 min$^{-1}$ | Antibiotic influx rate constant |
| $k_{out}$ | 0.01 min$^{-1}$ | Antibiotic efflux rate constant |
| $n$ | 1 | Hill coefficient for target synthesis term |
| $T_0$ | 1e-1 μM | Initial target concentration |
| $P_0$ | 0 μM | Initial antibiotic-target complex concentration |

**Appendix Table S3. Descriptions of antibiotics used**

| Antibiotic | Class | Description |
|---|---|---|
| Streptomycin (Strep) | Aminoglycoside | Protein synthesis inhibitor, binds to 30S ribosomal subunit |
| Gentamicin (Gent) | Aminoglycoside | |
| Spectinomycin (Spec) | Aminoglycoside | |
| Tetracycline (Tet) | Aminoglycoside | |
| Carbenicillin (Carb) | β-lactam | Cell wall synthesis inhibitor |
| Pencillin G (PenG) | β-lactam | |
| Cefotaxime (Cef) | β-lactam | |
| Ciprofloxacin (Cipro) | Fluoroquinolone | DNA synthesis inhibitor, binds to DNA gyrase |
| Chloramphenicol (Cm) | Amphenicol | Protein synthesis inhibitor, binds to 50S ribosomal subunit |

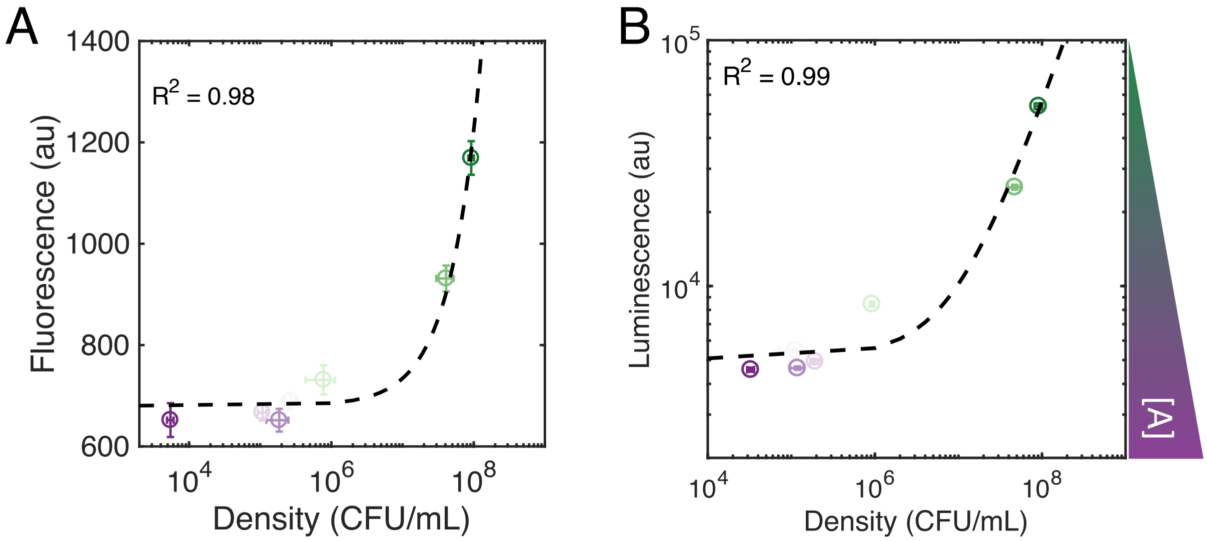


**Appendix Figure S1. Fluorescence luminescence reporters accurately reflect density following antibiotic treatment.**

A. The total GFP signal of a population increases approximately linearly with the cell density (as measured by CFU counts) after 2-hour antibiotic treatment. Here, colors indicate increasing streptomycin concentrations (0, 2, 4, 6, 8, 10, 12 μg/mL). Dashed line indicates linear fit; all error bars show the standard deviation of four replicates.

B. The total luminescence of a population increases approximately linearly with the cell density (as measured by CFU counts) after 2-hour antibiotic treatment.

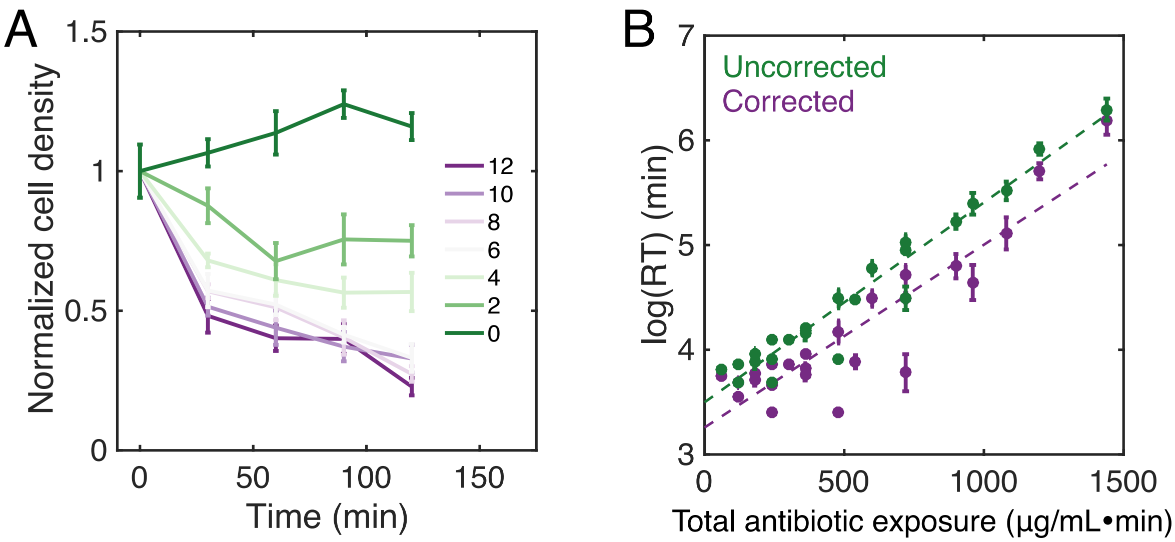


**Appendix Figure S2. Partial population death does not account for antibiotic dependent recovery time.**

A. Concentration dependent streptomycin killing rates were determined in the absence of population growth. Overnight cultures were diluted 10-fold and grown in M9 media to a high density. Cells were then incubated in minimal media with the indicated antibiotic concentration for increasing durations. Population viability decreased exponentially with dose duration. The control population showed negligible growth/death in these conditions, indicating that decrease in viability is due to cell killing. All data points show mean and standard deviation of 6 replicates; legend shows streptomycin concentrations in μg/mL.

B. Correcting for partial population death results in a lower effective recovery time (purple data points). However, the log-linear dependence of the recovery time on the total antibiotic exposure remains.

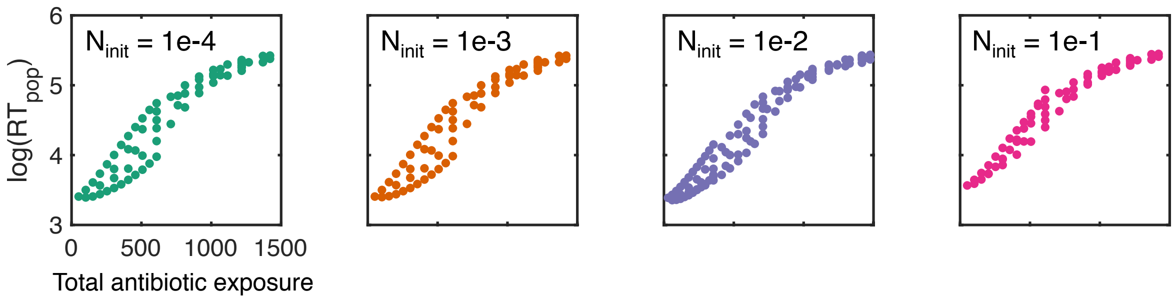


**Appendix Figure S3. Recovery time is not dependent on initial cell density.** Panels show recovery time in response to the same set of dosing conditions, with increasing initial cell densities. The relationship between recovery time and total antibiotic is maintained regardless of initial density.

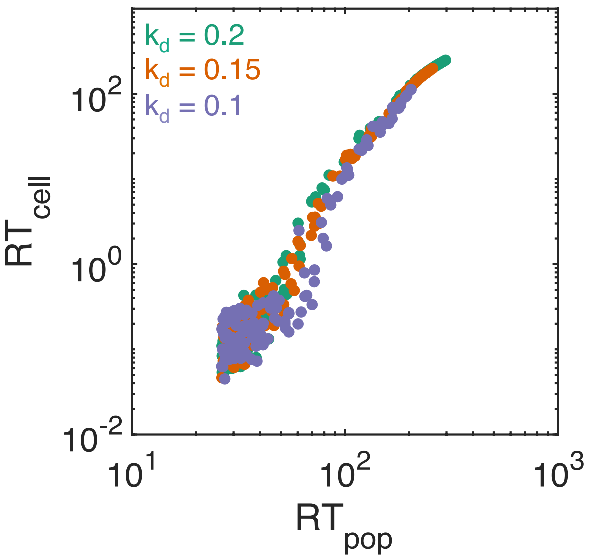


**Appendix Figure S4. Correlation between $RT_{pop}$ and $RT_{cell}$ is robust to ribosome degradation rate.** Individual and population recovery time are strongly correlated over a range of ribosome degradation rates. Here, $R^2$>0.8 in all cases.

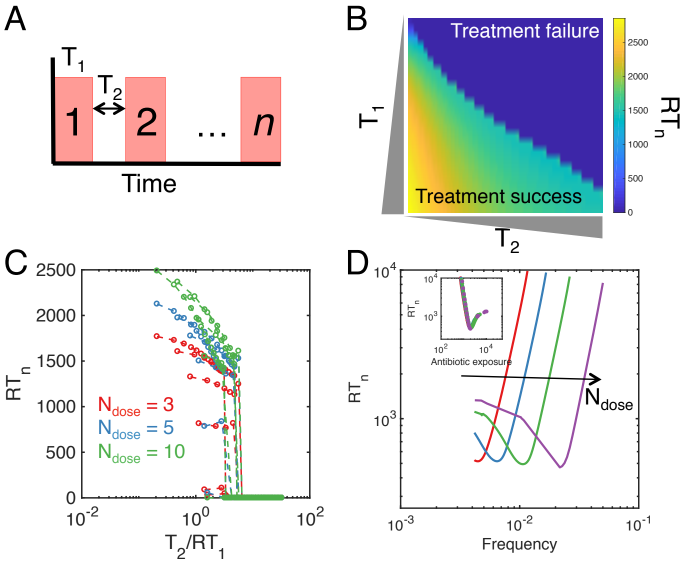


**Appendix Figure S5. Recovery time as a predictive metric for periodic dosing efficacy.**

A. Periodic dosing regimens can be specified by four key parameters: the antibiotic concentration $A$, the dosing interval $T_1$, the time between doses $T_2$, and the total number of doses, $N_{dose}$. We denote the recovery time following $n$ doses as $RT_n$.

B. Dosing parameters dictate treatment efficacy. Here, the heat map colors indicate recovery time after $N_{dose} = 10$, for each combination of $T_1$ and $T_2$; yellow corresponds to large recovery time (i.e. a successful treatment) and blue corresponds to a low recovery time (i.e. treatment failure).

C. $RT_1$ predicts the efficacy of $n$-dose treatments. Here, each trajectory corresponds to one value of $T_1$, and colors correspond to increasing values of $N_{dose}$; each data point corresponds to a single multi-dose treatment at a fixed antibiotic concentration $A$. There is a sharp transition in treatment efficacy (as measured by $RT_n$) at roughly $\frac{T_2}{RT_1} = 1$; that is, the recovery time in response to a single dose corresponds to the maximum value of $T_2$ that results in a successful multi-dose treatment.

D. $RT_n$ is a function of total antibiotic exposure. Multi-dose recovery exhibits a biphasic response to dosing frequency, wherein populations are able to recover faster at intermediate frequencies, regardless of the total number of doses (black arrow). Here, we assume that $T_1 = T_2$ for all doses, and thus frequency $f = \frac{1}{2T_1}$.

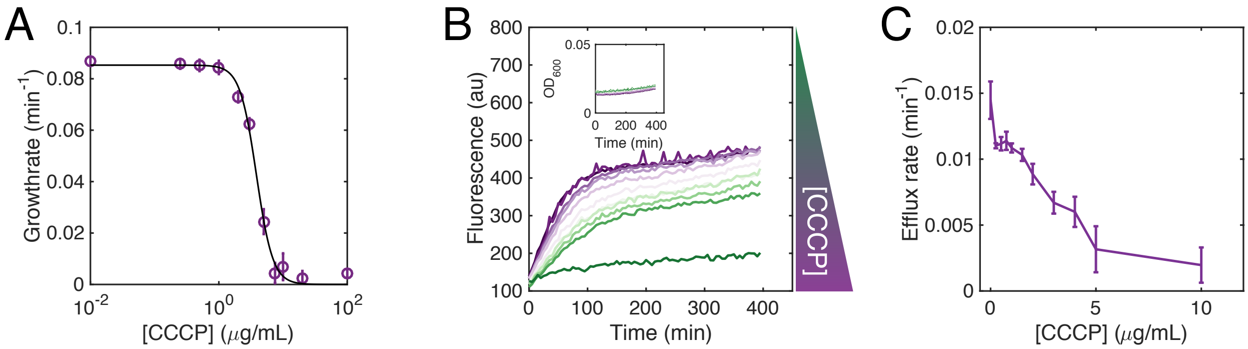


**Appendix Figure S6. [CCCP] inhibits efflux pump activity in a dose-dependent manner.**

A. Dose response for carbonyl cyanide m-chlorophenyl hydrazine (CCCP). Seven logarithmically spaced concentrations were chosen, in addition to 0. Growth rates were calculated by log-transforming time series data and calculating the slope of the longest linear segment. Data points are the average of three replicates; black line shows the best fit Hill function ($IC_{50}$ = 3.87μg/mL).

B. CCCP increases ethidium bromide (EtBr) accumulation, and inhibits antibiotic efflux, in a dose-dependent fashion. A previously published protocol was used to measure the effect of CCCP on efflux pump activity, in the absence of growth. Intracellular EtBr, which acts as a general efflux pump substrate, can be measured via population fluorescence; accumulation is increased in the presence of increasing CCCP concentrations.

C. Efflux rate decreases with the addition of CCCP. Rates were calculated by fitting fluorescence time series data to Equation (3). Data points are the average of eight replicates.

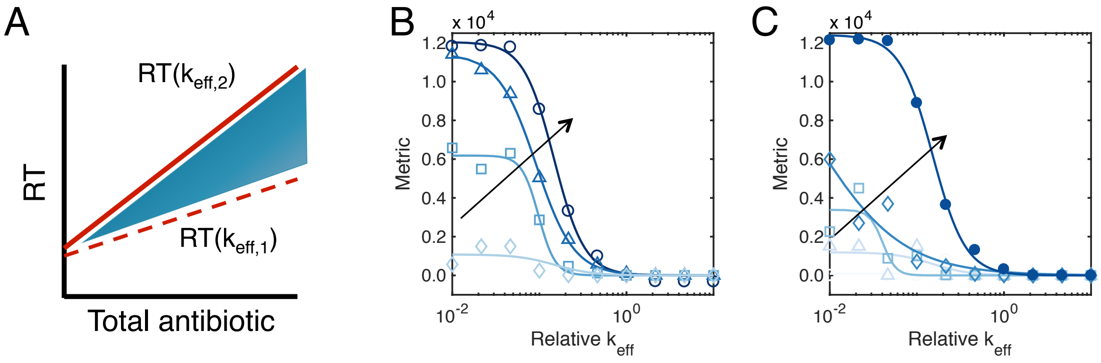


**Appendix Figure S7. Efflux inhibition is effective at high degradation rates and strong positive feedback conditions.**

A. Sensitivity is defined as the total change in recovery time in response to a change in the antibiotic efflux rate (e.g. $\boldsymbol{k_{eff,2}}$ vs $\boldsymbol{k_{eff,1}}$), over the same range of antibiotic treatments (shaded triangle). Larger changes indicate increased sensitivity, and smaller changes indicate minimal sensitivity.

B. Populations are more sensitive to decreases in antibiotic efflux rate in conjunction with rapid ribosome degradation. Sensitivity was quantified relative to the basal value of $\boldsymbol{k_{eff}}$, for increasing values of the ribosome degradation (arrow shows direction of increasing $\boldsymbol{k_d}$).

C. Populations are more sensitive to decreases in antibiotic efflux rate in conjunction with strong nonlinearity in positive feedback. Sensitivity was quantified relative to the basal value of $\boldsymbol{k_{eff}}$, for increasing values of the ribosomal feedback strength (arrow shows direction of increasing Hill coefficient $\boldsymbol{n}$).

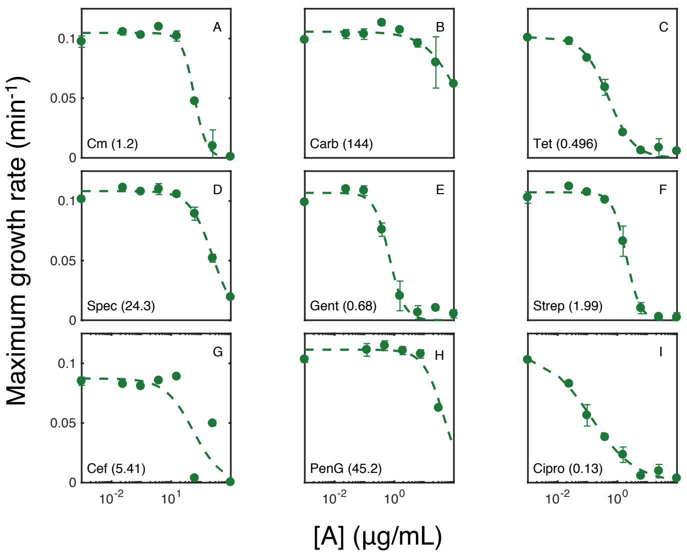


**Appendix Figure S8. Determining $IC_{50}$ values for various antibiotics.** $IC_{50}$ values were calculated from long-term dose responses in 96-well plates. Antibiotics were serially diluted to create a concentration gradient; cells were initially diluted 100-fold from overnight culture (~2e7 CFU/mL). Growth curves were collected using a Tecan M200 plate reader measuring $OD_{600}$ every ten minutes. Each data point corresponds to the average of three technical replicates. $IC_{50}$ values are shown in parentheses and were determined by fitting growth rates to a Michaelis-Menten equation (dashed line), and are shown in each panel (μg/mL).  Antibiotics tested are as follows: chloramphenicol (panel A), carbenicillin (B), tetracycline (C), spectinomycin (D), gentamicin (E), streptomycin (F), cefotaxime (G), penicillin G (H), and ciprofloxacin (I). For all panels, x-axis indicates antibiotic concentration on a log scale.
